# Outcomes of a rapid refeeding protocol in Adolescent Anorexia Nervosa

**DOI:** 10.1186/s40337-015-0047-1

**Published:** 2015-03-25

**Authors:** Sloane Madden, Jane Miskovic-Wheatley, Simon Clarke, Stephen Touyz, Phillipa Hay, Michael R Kohn

**Affiliations:** Eating Disorder Service, The Sydney Children’s Hospitals Network, Westmead Campus, Locked Bag 4001, Westmead, 2145, NSW Australia; Discipline of Psychiatry, Faculty of Medicine, The University of Sydney, Sydney, Australia; Westmead Clinical School, The Sydney Children’s Hospitals Network, Westmead Campus, Sydney, Australia; Discipline of Paediatrics, Faculty of Medicine, University of Sydney, Sydney, Australia; Centre for Research into AdolescentS’ Health (CRASH), University of Sydney, Sydney, Australia; Clinical Psychology Unit, University of Sydney, Sydney, Australia; School of Medicine, University of Western Sydney, Sydney, Australia

**Keywords:** Anorexia Nervosa, Eating disorder, Inpatient treatment, Refeeding syndrome, Adolescents, Nasogastric refeeding, Rapid refeeding

## Abstract

**Background:**

The impact of severe malnutrition and medical instability in adolescent Anorexia Nervosa (AN) on immediate health and long-term development underscores the need for safe and efficient methods of refeeding. Current refeeding guidelines in AN advocate low initial caloric intake with slow increases in energy intake to avoid refeeding syndrome. This study demonstrates the potential for more rapid refeeding to promote initial weight recovery and correct medical instability in adolescent AN.

**Methods:**

Seventy-eight adolescents with AN (12–18 years), hospitalised in two specialist paediatric eating disorder units, for medical instability (bradycardia, hypotension, hypothermia, orthostatic instability and/or cardiac arrhythmia) were followed during a 2.5 week admission. Patients were refed using a standardised protocol commencing with 24–72 hours of continuous nasogastric feeds (ceased with daytime medical stability) and routine oral phosphate supplementation, followed by nocturnal feeds and a meal plan of 1200-2400 kcal/day aiming for a total caloric intake of 2400–3000 kcal/day. Along with indicators of medical stability, weight, phosphate and glucose levels were recorded.

**Results:**

All patients gained weight in week one (*M* = 2.79 kg, *SD* = 1.27 kg) and at subsequent measurement points with an average gain of 5.12 kg (*SD* = 2.96) at 2.5 weeks. No patient developed hypophosphatemia, hypoglycaemia, or stigmata of the refeeding syndrome.

**Conclusions:**

The refeeding protocol resulted in immediate weight gain and was well tolerated with no indicators of refeeding syndrome. There were no significant differences in outcomes between the treatment sites, suggesting the protocol is replicable.

**Trial registration:**

Australian Clinical Trials Register number: ACTRN012607000009415

## Background

The impact of severe malnutrition in adolescent Anorexia Nervosa (AN) on short-term health and long-term growth demands safe and efficient means for nutritional and weight recovery [1]. This is particularly so in adolescents with medical instability (bradycardia, hypotension, hypothermia, orthostatic instability and cardiac arrhythmia) [2,3]. Increasingly, such patients are admitted to paediatric wards for medical stabilisation, initiation of refeeding and eating disorder treatment [4]. Current refeeding guidelines are inconsistent, lacking in empirical evidence, and do not adequately address physiological requirements for children and adolescents with AN [5].

Over the last two decades, guidelines for the nutritional management of AN have been published by a range of professional organisations including the Society of Adolescent Health and Medicine [3]; the National Institute for Clinical Excellence [6] and the American Psychiatric Association [1]. These current guidelines recommend an initial refeeding rate of between 10 and 40 kcal/kg per day or between 20-80% of total daily requirements, with slow caloric increases. Debate is ongoing as to the recommended rate of weight gain in inpatient settings with targets ranging from 500 to 1,400 g per week [1,7]. In all cases, the initial caloric prescription is below daily resting energy requirements with only slow increases recommended.

Current refeeding guidelines aim to avoid the refeeding syndrome principally by restricting the intake of calories. The refeeding syndrome is the occurrence of hypophosphatemia, cardiac events, neurological concerns and/or sudden death of patients within two weeks of commencing refeeding [8-15]. Despite the intent, current guidelines have not eliminated the occurrence of refeeding syndrome. A review of the literature by O’Connor and Goldin [14] identified 23 patients who developed refeeding syndrome despite slow rates of refeeding. The average rate of refeeding in these patients was 27 kcal/kg/day, well below total daily energy requirements. Of note, all patients were less than 70% of expected body weight for age, height and gender and all developed hypophosphatemia. They concluded individuals treated according to current guidelines might still develop the refeeding syndrome, with the risk not linked to caloric intake.

Literature exploring the physiology underlying the refeeding syndrome, reviewed by Kohn*,* Madden and Clarke [8], highlighted disturbances of insulin and phosphate as central to this process. The refeeding syndrome is thought to be instigated by the influx of enteral glucose during refeeding causing an insulin surge and driving glucose, fluid and electrolytes into the intracellular space. This combined with the breakdown of the sodium/potassium pump regulating the intracellular environment due to a lack of phosphated adenosine to provide energy to the pump results in severe electrolyte disturbances, hypophosphatemia, cardiac failure and death [14]. Based on this understanding, it has been argued that restricting the percentage of daily energy provided by carbohydrate rather than total daily energy intake, in addition to the provision of supplemental phosphate, may be more effective in mitigating the risk of refeeding syndrome than reducing calories [8].

The starvation state is associated with low basal levels of insulin and high glucagon levels. It is hypothesised that this results in a delayed insulin response to refeeding, resulting in clinically significant post-prandial hypoglycaemia during early refeeding [14,16-19]. A study by Hart et al., [20] reported that 29% of adult patients with AN experienced post-prandial hypoglycaemia in the first weeks of refeeding with the highest rates occurring between one and three hours post meal. In 2011, Kohn et al., [8] proposed the use of continuous nasogastric refeeding during early refeeding to eliminate hypoglycaemia.

Clinical settings using current feeding guidelines have reported poor weight gain or weight loss in hospitalised adolescents with AN in the first week of admission resulting in further deterioration in nutritional status [21]. Six recently published studies, four retrospective chart reviews and two prospective cohort studies [15,21-25] have concluded that higher initial refeeding rates lead to faster weight gain, more rapid medical stabilisation and shorter lengths of hospital stay without adverse consequences.

The aim of the current study is to report refeeding outcomes for a cohort of adolescent patients with AN using a standardised refeeding regime involving supported oral meals and nasogastric feeding with sufficient calories to initiate and sustain weight gain from the outset of treatment. The proposed refeeding protocol aimed to address the risks associated with both the refeeding syndrome, poor weight gain and delayed medical recovery by limiting energy provided as carbohydrate to a maximum of 50% while providing sufficient energy to exceed total daily energy requirements. All patients were commenced on oral phosphate supplementation (500 mg Sandoz phosphate, twice daily). Additionally, the current protocol was designed to eliminate post-prandial hypoglycaemia through continuous nocturnal NG feeds, a practice well tolerated by patients [26].

We hypothesized that the presented protocol would be well tolerated, promote timely weight restoration and not result in the occurrence of the refeeding syndrome.

## Methods

**Figure 1:**
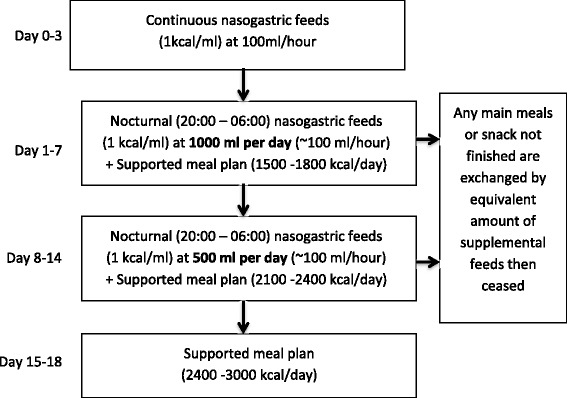
**Refeeding protocol used in the nutritional rehabilitation of patients.**

This study was carried out in two specialist paediatric eating disorder services located in adjacent university teaching hospitals (The Sydney Children’s Hospital Network, Westmead Campus (SCHN-W) and Westmead Hospital (WH)). A standard refeeding protocol, described in Figure 1, was followed at both treatment sites and compared to assess reliability.

### Participants

This study cohort included all patients in a large randomised controlled inpatient treatment trial [27]. Participants were recruited from 266 consecutive eating disorder admissions to two specialist paediatric medical units between June 2007 and February 2010. Participants were eligible if they were aged between 12 and 18 years, with a DSM-IV diagnosis of AN of less than 3 years duration [28] and were medically unstable (hypothermic (temperature <35.5°C), bradycardic (heart rate < 50 beats/min), hypotensive (blood pressure < 80 mm Hg systolic and < 40 mm Hg diastolic), orthostatic instability (pulse increase > 20 beats/min, systolic blood pressure decrease > 20 mm Hg) or QT interval corrected for heart rate > 0.45 s) [3]. All participants subsequently would have met DSM 5 criteria for AN [29]. Eighty-five patients met trial criteria and 82 patients and their families consented to the research. Four patients withdrew from the trial during inpatient treatment, leaving 78 patients completing the inpatient treatment refeeding protocol at a 95% retention rate.

### Assessments and procedures

Medical assessments were conducted by paediatricians experienced in the management of eating disorders and psychiatric assessments by an experienced child psychiatrist. Assessment included diagnostic evaluation for eating disorder symptomatology using medical and psychiatric assessment and standardised clinical interviews and questionnaires including the Eating Disorder Examination adult, youth and child versions [30-32].

### Medical assessment

Body weight was recorded at intake, then bi-weekly for the duration of the admission. Body weight was recorded to the nearest 0.05 kg using a platform scale (A&D FV-150 K) before breakfast, post-voiding, in a hospital gown and without footwear. Height was measured to the nearest 0.1 cm with a stadiometer (TB I Hyssna AB). Percent Expected Body Weight (%EBW) was calculated using the 50^th^ percentile on tables for weight and height by age and gender using the Centre for Disease Control and Prevention standardised growth charts [33] (%EBW = BMI/50^th^ percentile BMI for age, height and gender × 100) [34].

To assess medical stability, heart rate (per minute) was reported as three times the recorded value for radial pulse measured for 20 seconds. Temperature was taken using a Becton and Dickinson electronic thermometer placed in the axilla. Blood pressure was recorded using a cuff covering two-thirds of the length of the right arm connected to a Space Lab Medical SL electronic sphygmomanometer. Participants were initially placed on 24 hour continuous cardiac monitoring with pulse rate, blood pressure and temperature recorded two-hourly. Once criteria for medical instability were no longer met during the day, study participants had four-hourly monitoring of pulse rate, blood pressure and temperature monitoring during the day, but remained on continuous overnight monitoring between 22:00 and 06:00 hours. Patients were examined on a daily basis for complications of refeeding syndrome including cardiac failure, dependent oedema and delirium.

Morning blood samples were taken to track blood electrolytes including phosphate and blood glucose levels. Blood samples were taken daily for the first 7 days and then twice weekly. Urinalysis, looking at specific gravity as a measure of hydration, was taken at admission and bi-weekly in conjunction with weights for the duration of admission.

### Measures

The primary outcome measure was change in %EBW at week 1, week 2 and 2.5 weeks. Secondary outcome measures included hypophosphatemia, hypoglycaemia, electrolyte abnormalities and clinical stigmata of the refeeding syndrome (cardiac failure, dependent oedema and delirium).

### Treatment

Patients were refed using a standardised protocol commencing with 24 to 72 hours of continuous nasogastric feeds, ceased with day time medical stability, followed by a combination of nocturnal feeds and an oral meal plan aiming for a total caloric intake of between 2400–3000 kcal/day. Nasogastric feeds were limited to ≤50% of energy from carbohydrates with approximately 30% energy from fats. Patients were prescribed routine phosphate supplements (Sandoz Phosphate, 500 mg, twice daily) from the commencement of treatment. The amount and duration of nasogastric feeding was determined by markers of medical instability. The total amount of prescribed calories was calculated to result in a weekly weight gain of 1 kg (see Figure 1). Fluid intake was limited to that provided by the nasogastric feeds and that prescribed in the oral menu plan and varied between 1540 ml and 1920 ml per day.

### Ethical approval

This study was approved by the Human Research Ethics Committee of the Sydney Children’s Hospital Network, Westmead Campus, and Westmead Hospital (2006/114). Written consent was obtained from all participants and their families at hospital admission.

### Statistical analysis

To assess group differences between the two treatment sites, independent t-tests were used. Repeated-measures analysis of variance was used to compare weight across time points. The criterion used for statistical significance was *p* < 0.05 in two-tailed tests. Analyses were performed using SPSS version 21 for Mac.

## Results

Fifty-two participants (67%) were treated at SCHN-W and 26 participants (33%) at WH. Participants were mostly female (95%), aged 12–18 years with a mean age of 14.84 years (*SD* = 1.46). The only statistically significant difference on baseline variables between sites was age at admission [SCHN-W (*M* = 14.00, *SD*=. 99) vs. WH (*M* = 16.17, *SD* = 1.05, *t*(76) = −9.24, *p* < .05) which was to be expected due to the different admission age criteria for each unit. There were no differences in illness history, clinical factors, or length of inpatient treatment between groups (Table 1). The protocol was consistently adhered to by all participants included in the analysis.

**Table 1:** **Demographic and clinical characteristics of sample**

	**N (%)** ^**a**^ **or** ***M (SD)*** ^***b***^		
Demographic characteristics			
	**SCHN-W**	**WH**	**Total**
Sample Size	52	26	78
Age (years)*	*14.00 (.99)*	*16.17 (1.05)*	*14.84 (1.46)*
Gender - Male	1 (1.92)	3 (11.54)	4 (5.13)
Ethnicity			
White	43 (82.69)	22 (84.62)	65 (83.33)
Asian	9 (17.31)	0	9 (11.54)
Other	0	4 (15.38)	4 (5.13)
Clinical characteristics at admission			
Anorexia Nervosa subtype			
Restricting only	36 (69.23)	17 (65.38)	53 (67.95)
Restricting with bulimic features	16 (30.77)	9 (34.62)	25 (32.05)
Excessive exercise	19 (36.54)	10 (38.46)	29 (37.18)
Duration of illness (months)	*6.56 (4.37)*	*9.27 (7.97)*	*7.60 (6.16)*
Previous hospitalizations	4 (7.69)	1 (3.85)	5 (6.41)
Percent expected body weight	*78.88 (6.13)*	*77.54 (7.09)*	*78.37 (6.50)*
Eating disorder examination global score	*3.04 (1.12)*	*2.99 (1.13)*	*3.02 (1.12)*
Treatment characteristics			
Length of admission (days)	*30.27 (16.24)*	*25.97 (13.46)*	*28.62 (15.28)*

^a^Chi-square test for independence was conducted to compare group differences for categorical variables and no statistically significant differences were found at the .05 level. ^b^Independent-samples t-test was conducted to compare trial group mean differences for each continuous variable and no statistically significant differences were found at the .05 level unless otherwise noted. *Statistically significant difference at the *p* < .05 level.

The mean %EBW on admission was 78.37 (*SD* = 6.50). Paired-samples t-test revealed that there was a statistically significant gain in weight from admission to the end of the refeeding protocol for the cohort as a whole, with %EBW at 2.5 weeks on average 85.58 [*SD* = 6.54, *t*(77) = −17.02, *p* < 0.05, eta squared = .79 (very large effect)]. Weight gain in the first week of admission was at a mean of 2.79 kg (*SD* = 1.27) with gains continuing throughout the 2.5 week admission (*M* = 5.12 kg, *SD* = 2.96). Statistically significant weight gain was seen from the end of the refeeding protocol to 12-month post-treatment follow-up where %EBW was 95.33 [SD = 9.47, t(77) = −14.30, *p* < 0.05, eta squared = .73 (very large effect)].

Although there was a significant difference in weight (kg) gained between sites at 0.5, 1, 1.5, 2 and 2.5 weeks, when weight was related to age, gender and height (i.e., transformed to %EBW), there was no significant difference in changes in %EBW between sites (see Table 2). The significant difference in weight gain when measured in kilograms relates to the significant difference in patient age between the two services. There was no significant difference in weight gain measured in kilograms based on AN subtype (Restricting: *n* = 54; *M* = 3.88, *SD* = 1.95; Binge/Purge: *n* = 24, *M* = 3.45, *SD* = 1.95; *t*(77) = .915, *p* = .363, NS).

**Table 2:** **Weight change over the course of admission**

	***M (SD)***						
	**%EBW per assessment**				**Weight gain per assessment (kg)**		
	**SCHN-W**	**WH**	**Total**		**SCHN-W**	**WH**	**Total**
Sample size	52	26	78	Sample size	52	26	78
Admission	*78.88 (6.13)*	*77.54 (7.09)*	*78.37 (6.50)*	Admission Weight	39.57 (4.89)	43.26 (6.30)	40.99 (5.72)
Week 0.5	*82.93 (6.98)*	*80.13 (6.66)*	*81.85 (6.95)*	Week 0.5*	*2.01 (1.31)*	*1.42 (.83)*	*1.78 (1.78)*
Week 1	*84.80 (6.45)*	*82.19 (6.59)*	*83.80 (6.59)*	Week 1	*.93 (.92)*	*1.14 (.60)*	*1.01 (.81)*
Week 1.5	*85.62 (6.51)*	*83.42 (6.32)*	*84.77 (6.49)*	Week 1.5*	*.41 (.54)*	*.68 (.64)*	*.51 (.59)*
Week 2	*85.88 (6.66)*	*84.08 (6.28)*	*85.19 (6.54)*	Week 2*	*.13 (.45)*	*.36 (.51)*	*.22 (.48)*
Week 2.5	*85.90 (6.49)*	*85.06 (6.48)*	*85.58 (6.46)*	Week 2.5*	*.01 (.62)*	*.54 (.683)*	*.22 (.62)*
Total Gain	*7.02 (3.21)*	*7.52 (4.42)*	*7.21 (3.70)*	Total Gain	*4.98 (2.37)*	*5.34 (3.75)*	*5.12 (2.96)*
12 month FU	*94.90 (10.21)*	*93.94 (7.87)*	*94.53 (9.33)*	*12 month FU Weight*	*51.59 (5.79)*	*56.00 (7.44)*	*53.00 (6.64)*

Independent-samples t-test was conducted to compare trial group mean differences for each continuous variable and no statistically significant differences were found at the .05 level unless otherwise noted. *Statistically significant difference at the p < .05 level.

**Figure 2:**
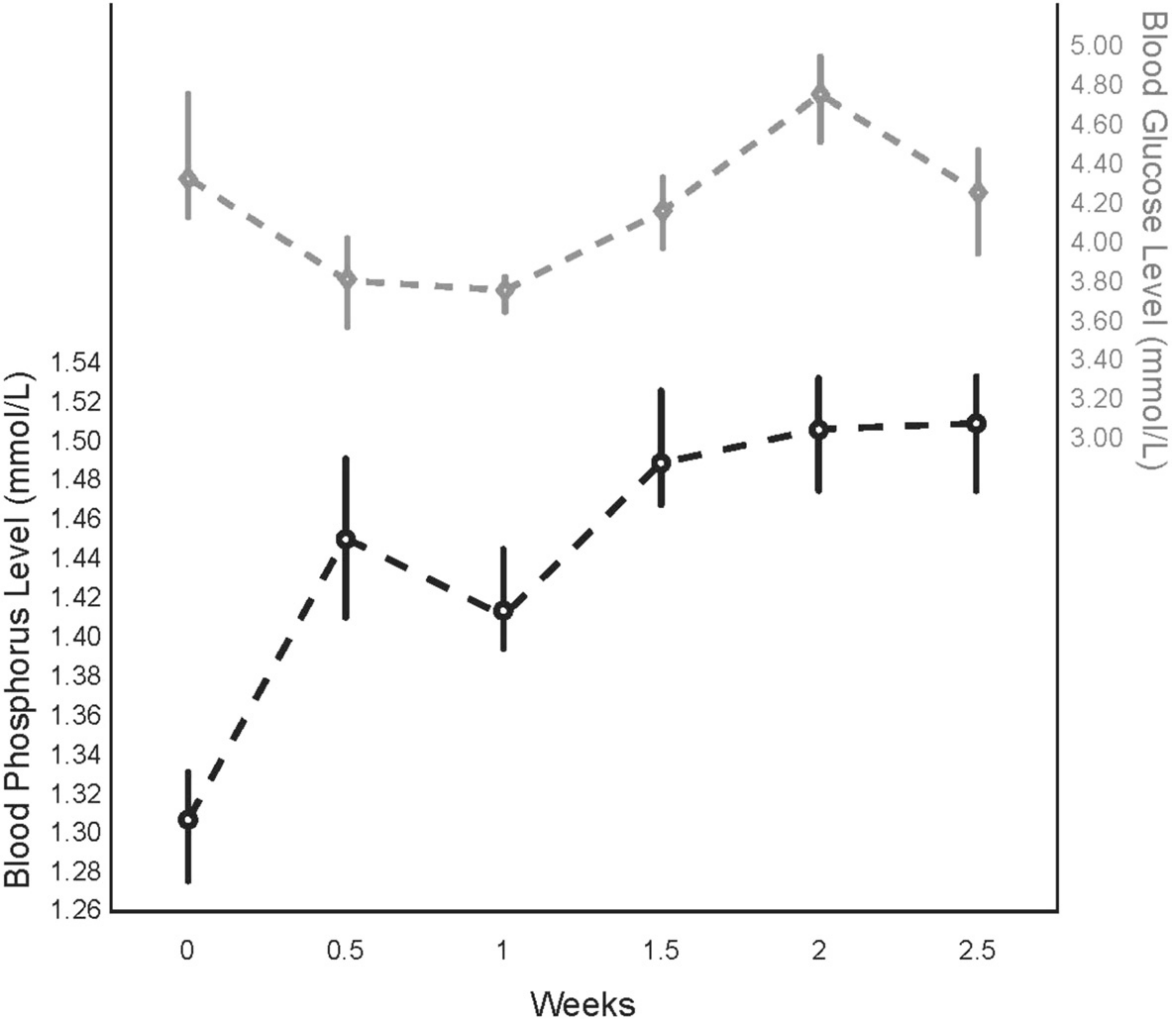
**Blood levels with weight gain.**

Serum phosphate and blood glucose levels were measured daily for a minimum of 7 days and then twice weekly once stable. No patient developed hypophosphatemia (<1.0 mmol/L), or hypoglycaemia (<3.0 mmol/L) at any point during treatment (see Figure 2).

All patients were examined medically on a daily basis during the course of their admission. No patient had an adverse reaction to the refeeding protocol nor developed any clinical signs or symptoms of the refeeding syndrome. In particular no patient developed signs of cardiac failure, dependent oedema or delirium.

## Discussion

The refeeding guidelines proposed by this study (Figure 1) promote the provision of sufficient caloric intake to meet metabolic needs and facilitate weight gain from the outset of refeeding. The proposed starting caloric intake and rate of increase in caloric prescription exceeds that of current guidelines [1,3,6]. Patients treated with the study refeeding protocol safely and significantly gained weight over the course of treatment including in the first week of admission, without incidence of refeeding syndrome (cardiac failure, dependent oedema or delirium) or other complications, including hypophosphatemia or post-prandial hypoglycaemia. In addition to early weight gain the group showed ongoing weight gain to 12-month follow up with average %EBW (95.33) consistent with definitions of weight recovery [35].

Weight gain within the first week of refeeding averaged 2.79 kg. While this rate of weight gain is significantly in excess of what would be expected based on the prescribed caloric intake and in excess of current guideline recommendations [1,7], with ongoing nutritional rehabilitation, weight gain averaged 1.34 kg per week during the first 2.5 weeks of admission. This was consistent with the theoretical increase from the total calories provided and was consistent with previous outcomes for patients receiving 2700 – 3000 kcal each day [15]. All patients had base-line body composition studies using dual x-ray absorptiometry (DXA), however, radiation exposure associated with this method precluded its use to measure changes in body composition over the period of this study. Previous research by our group has shown that other methods for measuring body composition (bioelectrical impedance and skin fold thickness) to be inaccurate on an individual level and with wide levels of variation between results obtained for different methods [36,37] and for this reason were not used to measure changes in body composition over this period. Physical examination and regular urinalysis did not reveal evidence of over-hydration in this group but it is likely that a portion of the weight gain in week one was due to rehydration. Research has shown that reestablishment of glycogen stores in the liver may account for significant weight gain with glycogen itself accounting for 400 g in healthy adults [38] and water bound to glycogen up to 1.2 kg [39].

The early weight gain in this study contrasts with treatment centres following currently published guidelines who universally report a decrease in weight during the first week of refeeding [21]. In the case of malnourished AN patients presenting with medical instability, the provision of insufficient calories to meet resting energy expenditure has been shown to prolong medical instability and the length of hospitalisation in this patient group [15,21-25]. There was no significant difference in weight gain between treatment sites when weight was related to gender, age and height, suggesting the replicability of this protocol.

Clinically significant post-prandial hypoglycaemia during initial refeeding of patients with AN has been reported in the literature [14,16-18]. In the current study, continuous nocturnal nasogastric tube feeding was used to address post-prandial hypoglycaemia by maintaining a constant level of available calories to maintain blood glucose levels and promote the restoration of tissue glycogen. Though it was beyond the scope of this study to systematically measure post-prandial blood glucose levels at all meals, no hypoglycaemia was observed in morning blood glucose measurements, taken 60–150 minutes after breakfast.

Avoidance of carbohydrate as the predominant macronutrient energy source was undertaken in view of previous observations that high carbohydrate loads predispose to hypophosphatemia and the refeeding syndrome [14]. The proposed protocol limited the proportion of carbohydrates to <50% of dietary energy intake. In addition, oral phosphate supplements (500 mg, twice daily) were given during the study period. Hypophosphatemia was not seen in this patient group.

Psychological outcomes in this group have been previously published demonstrating low patient drop out rates during inpatient refeeding (4 out of 82 patients (4.9%)) and significant improvements in eating disorder psychopathology at 12-month follow-up as measured by Eating Disorder Examination global score (Admission: M = 3.03, SD = 1.13 vs. 12 month follow up: M = 1.59, SD = 1.36, t(68) = 7.88, *p* < 0.05, eta squared = .49 (very large effect)) [27]. Research published by this group has shown this protocol, in particular nasogastric refeeding, to be well tolerated in adolescents with AN [26] These findings are consistent with published research which has not demonstrated negative psychological outcomes with more rapid weight gain associated with nasogastric refeeding [40], high energy supplements [41] and oral refeeding [42] including weekly rates of weight gain in excess of 2 kg [42].

The protocol developed for this study arose from the need to have a standardised treatment regimen for this multi-centre randomised controlled inpatient treatment trial of medically unstable adolescents with AN [27]. The refeeding protocol was developed to better meet physiological need, to improve safety, and avoid refeeding syndrome. The described protocol provides important details, directions and outcomes about refeeding medically compromised adolescents with AN more rapidly than in current guidelines [1,3,6]. These results suggest that calorie prescription can safely exceed levels recommended in contemporary guidelines.

## Conclusions

The refeeding protocol resulted in immediate weight gain and was tolerated with no indicators of refeeding syndrome. There were no significant differences in outcomes between the treatment sites, suggesting the protocol is replicable. The experience of using this refeeding protocol provides support for reviewing current recommendations for the treatment of medically unstable adolescents with Anorexia Nervosa.

## Abbreviations

AN: Anorexia nervosa
SCHN-W: The Sydney children’s hospital network (Westmead Campus)
WH: Westmead hospital
%EBW: Percent expected body weight
